# Seeing the disease before it has manifested itself

**DOI:** 10.1007/s12471-019-1236-3

**Published:** 2019-01-29

**Authors:** Y. Pinto

**Affiliations:** Amsterdam University Medical Centre, University of Amsterdam, Amsterdam, The Netherlands

In this issue of the Journal, van Velzen et al. [1] report on indices of cardiac function in subjects with hypertrophic cardiomyopathy, with special attention to longitudinal strain.

Hypertrophic cardiomyopathy is, among the cardiomyopathies, rather common with an estimated prevalence of 1:500. The disease is defined by its clinical appearance of asymmetric cardiac hypertrophy so that the diagnosis initially relied on clinical signs related to the hypertrophy as seen on echocardiography or electrocardiography. This changed in the early 1990s when the inherited nature of the disease allowed the identification of the underlying genetic variants that cause HCM, which in turn made it possible to identify carriers of an HCM causing mutation even before the HCM has manifested itself [2].

The ability to identify pre- or subclinical mutation carriers has an enormous impact, as it allows analysis of the disease without the need to wait for it to fully develop. This enables researchers to identify the earliest changes that occur in the myocardium well before full-blown hypertrophy is established. The course of events in HCM has thus yielded, rather surprisingly, that this cardiomyopathy may be related to enhanced rather than depressed function of the affected sarcomeres, myocytes and hence ventricle.

This notion is well illustrated in the study by van Velzen et al. [1]. They studied mutation carriers (mainly MyBPC and MYH7 mutations) at a stage at which traditionally HCM would not have been diagnosed, with a mean maximal wall thickness of 9 mm and only slight enlargement of the left atrium (on average 2 mm larger than in controls). However, some clear differences in cardiac function were already apparent, such as signs of diastolic dysfunction and increased global longitudinal strain (see their Table 2). Strikingly, despite a lack of severe hypertrophy they found increased ejection fraction and lower end-systolic volumes in the HCM mutation carriers. These data may suggest hypercontractility together with diastolic dysfunction.

As the authors discuss, there is conflicting data regarding these parameters in early stages of HCM. This most likely reflects regional differences within the myocardial wall in conjunction with differences in the phase of the disease at which it is assessed. Isolated sarcomeres that carry an HCM mutation have also been reported to demonstrate both depressed but also enhanced contractility [3].

Nevertheless, the current findings underscore that even before HCM has become clinically manifest, the underlying changes at the level of the sarcomere are already profound and may yield combined abnormalities in both systolic and diastolic function. How such analyses relate to patients is now becoming apparent. The basic finding of hypercontractility led researchers to postulate that in HCM it may actually be beneficial to inhibit sarcomeric function. As a result, the drug mavacamten (MYK-461), which inhibits sarcomere force production [4], has recently entered clinical testing in the PIONEER and PIONEER OLE study. The findings will show whether these early hypercontractile changes are indeed crucial and would provide a novel target for therapy.
